# Effect of heat treatment on mechanical properties and dimensional accuracy of 3D-Printed black carbon fiber HTPLA

**DOI:** 10.1016/j.heliyon.2024.e32282

**Published:** 2024-06-01

**Authors:** Ahmad Shah Hizam Md Yasir, Nor Aiman Sukindar, Ahmad Afif Abdul Rahman Putra, Yang Chuan Choong, Shafie Kamaruddin, Azlan Aziz, Yulfian Aminanda, Mohd Hafis Sulaiman

**Affiliations:** a Faculty of Resilience Rabdan Academy, 65, Al Inshirah, Al Sa'adah, Abu Dhabi, 22401, PO Box:114646, United Arab Emirates; b School of Design, Universiti Teknologi Brunei, Jalan Tungku Link Gadong BE1410, Brunei Darussalam; c Department of Manufacturing and Materials Engineering, International Islamic University Malaysia, 53100 Jalan Gombak, Kuala Lumpur, Malaysia; d Engineering Faculty, Universiti Teknologi Brunei, Jalan Tungku Link Gadong BE1410, Brunei Darussalam; e Department of Mechanical and Manufacturing Engineering, Faculty of Engineering, 43400 Serdang, Universiti Putra Malaysia

**Keywords:** 3D printing, black carbon fiber HTPLA, Dimensional accuracy, Taguchi method

## Abstract

This present study investigated how heat treatment affects the mechanical properties of 3D-printed black carbon fiber HTPLA by manipulating two parameters: heating temperature and holding time. The mechanical properties of 3D-printed black carbon fiber HTPLA components are crucial for assessing their structural integrity and performance. The shrinkage and dimensional accuracy of the 3D-printed parts were also explored using a vernier caliper. The microstructure of both heat-treated and non-heat-treated HTPLA black carbon fiber 3D-printed parts was examined using scanning electron microscopy. Samples were prepared, printed, heat-treated, and mechanically tested, and their microstructure was observed and recorded. The results showed that heat treatment improved the material's strength, hardness, and crystallinity, leading to better mechanical properties. However, statistical analysis indicates no clear evidence that the two factors, optimum heating temperature and holding time, affect the mechanical properties of heat-treated printed parts. Nonetheless, further study suggests that these factors might be important in optimizing the heat treatment process.

## Highlights

• Heat treatment mainly affects the mechanical properties of black carbon fiber HTPLA.
• Heat treatment improved the overall structure of HTPLA filament.
• The heat-treated HTPLA material has low dimensional accuracy, specifically on thin dimensions.
• Shrinkage differences of heat-treated printed parts are relatively small for all axes.

## 1 Introduction

The use of 3D-printing technology has become increasingly popular in recent years, allowing for creating complex and intricate designs with ease. 3D-printing technology is renowned for its flexibility and cost effectiveness. 3D printing or additive manufacturing is an engineering process used to produce 3D products layer-by-layer from a 3D-digital model as per required dimensions [1]. One of the most important factors affecting the mechanical properties of 3D-printed products is the microstructure, which is influenced by the porosity of the content and the distribution of pores. According to Rouf et al., 3D printing has evolved as a disruptive technology for the fabrication of industrial components [2]. The authors also noted that the technical and economic advantages of 3D printing make it a potential alternative to traditional manufacturing processes, especially when developing complex and optimized products. These statements highlight the potential of 3D printing to revolutionize the manufacturing industry and the need for further research to understand mechanical properties of 3D-printed products. The 3D-printed filament produced by the combination of carbon fiber and polylactic acid (PLA) is stiffer and stronger than that of conventional PLA [3]. According to Sanei and Popescu, carbon fibers substantially improve PLA's mechanical characteristics, increasing its resistance to fatigue and deformation. The addition of carbon fibers enhances the material's ability to endure heat at temperatures greater than those of standard PLA [4].

Black carbon fiber high-temperature polylactic acid (HTPLA) is distinguished by its unusual matte black appearance and rough surface. It is a type of 3D-printing material that is known for its strength and durability. Short carbon-fiber-reinforced HTPLA composites have lower interlayer tensile strength than the corresponding pure PLA in 3D-printed parts. This decrease in tensile strength of the composite is due to slower diffusion bonding between its adjacent layers due to an increase in melt viscosity caused by high heat resistance property of the carbon fiber [5]. Moreover, the mechanical properties and dimensional accuracy of 3D-printed materials can be affected by various factors, including heat treatment.

Unfortunately, the tribological properties of 3D-printed objects were inadequately understood by a recent study [2]. The infill pattern was designed according to the current universal plastic pattern printed at seven different angles in three thicknesses for each angle. Because the residual sum of squares between theoretical and experimental data is close to zero, the resulting theoretical model can accurately predict the tensile strength of fused deposition modeling (FDM) materials for all angles and thicknesses [6]. Studies on 3D-printed objects often prioritize optimizing printing settings rather than investigating processes that may permanently impact the microstructure. This gap in research hinders a comprehensive understanding of mechanical behavior, highlighting the need to explore long-term effects on 3D-printed structures for enhanced reliability.

Heat treatment involves heating a material to a specific temperature and holding it at that temperature for a certain amount of time. A study was conducted to investigate the impact of heat treatment and build orientation on the mechanical properties of 3D-printed parts, focusing on titanium alloy Ti6Al–4V. Various build orientations (0°, 45°, and 90°) and four heat treatment temperatures (0 °C, 700 °C, 800 °C, and 900 °C) were examined [7]. The results show that stress-strain or strain-strength relations in titanium alloy 3D-printed parts are positive only up to a certain temperature limit, beyond which stress reduces significantly. This limit is influenced by the object's size, shape, and physical properties.

Annealing can improve the internal structure and mechanical characteristics of 3D-printed objects made from PLA and HTPLA materials [5]. Heat treatment is an appropriate method to remove the porosity defect because the air gaps in the material are filled with itself when it melts at high temperatures during this process [8]. Previous studies discuss the performance of mechanical properties in 3D-printed parts using PLA filament, highlighting issues such as poor inter-filament bonding and residual thermal stresses. The study proposes heat treatment as a solution, noting that treating parts at 100 °C for 4 h can significantly enhance tensile properties by up to 80 % and improve heat resistance by 73 %, as measured by the heat distortion temperature test [9]. The failure mode of the matrix layers was changed, and the adhesion between the fiber bundles and the impregnated matrix was again enhanced by heat treatment [10]. This process can affect the microstructure of the material, which in turn can affect its mechanical properties and dimensional accuracy. The increase in the interlayer resistance is related to the interlayer diffusion of particles during annealing [11].

In addition, another study examined the effects of in-process annealing by manipulating printing direction, layer thickness, and temperature. The results indicate that heat treatment positively impacts tensile strength, while nozzle diameter and printing direction affect hardness [12]. The findings suggest the viability of in-process treatments for enhancing manufacturing processes, potentially leading to cost-effective and sustainable outcomes.

The 10.13039/501100014063FDM process struggled to produce fine structures without support [13]. The importance of optimizing process parameters and postprocessing operations has proven to boost the bonding quality between rasters and layers as well as minimize voids in printed products [14]. Heat treatment could enhance the mechanical properties of the material by reducing porosity and thereby strengthening the interface [15]. Most researchers have explored ways to enhance mechanical properties, ranging from remarkable improvements in tensile strength and heat resistance to optimization of continuous fiber-reinforced composites. The observations included the temperature-dependent effects on deformation for vertically- and horizontally-printed PLA specimens, effectiveness of annealing for interlayer tensile strength without modifications to the printing process, and the importance of optimizing nozzle temperature and heat treatment for improved bonding and minimized voids in printed products.

In this study, the effect of heat treatment on the mechanical properties and dimensional accuracy of 3D-printed black carbon fiber HTPLA was explored. Short carbon-fiber-reinforced HTPLA composites exhibit lower interlayer tensile strength compared to pure PLA in 3D-printed parts which have more brittleness due to crystallization with temperature changes, [5]. Therefore, this present study attempted to increase the mechanical properties of the black carbon fiber HTPLA printed parts by manipulating the holding temperature and heating time. The results were then analyzed, and the heated samples were compared with the unheated samples to assess their performance in terms of tensile strength, compressive strength, and flexural strength. ANOVA analysis was employed to determine the potential impact of the two variables on the mechanical properties. In addition, the study analyzed the shrinkage and dimensional accuracy of the heated black carbon fiber HTPLA. By examining the effect of heat treatment on this material, valuable insights were gained, such as how heat treatment can be used to improve the mechanical properties and dimensional accuracy of 3D-printed materials.

## 2 Materials and methods

### 2.1 Experimental setup

This study was conducted to analyze the surface roughness and dimensional accuracy of parts printed using FDM. Setting up the 3D printer and selecting the proper material for the intended use are both essential steps in the preparation of samples for 3D printing. In this case, the black carbon fiber composite HTPLA filament from PROTOPASTA, USA was printed using an Artillery Sidewinder X1(Artillery 3D, China) 3D printer. The infill density is chosen to be 100 %, which balances the printed pieces’ weight and strength. The control parameters for the printer are shown in Table 1.

**Table 1:** Recommended printing parameters by manufacturer for black carbon fiber composite HTPLA filament.

Printing Speed (mm/s)	50
Nozzle Diameter (mm)	0.8
Layer Thickness (mm)	0.2
Printing Temperature (°C)	200
Raster Angle (°)	0.0
Infill Density (%)	100 %
Bed Temperature (°C)	80
Z Seam Position	Tensile and Flexural - corner Compressive - random

These parameters ensure that the 3D model is made according to the best printing results, which is another crucial part of sample preparation. This crucial part also includes looking for any problems that can influence printing, such as nonmanifold geometry, crossing faces, or other mistakes. To ensure that a digital model is acceptable for 3D printing, it will be tested using Computer Aided Design software tools, such as UltiMaker CURA. Finally, it is essential to set up the print bed correctly before printing. To ensure that the printed part sticks to the bed and does not deform or detach during printing, an appropriate adhesive, such as glue or hairspray, must be applied. To guarantee that the first layer of the print is firmly attached to the bed and that the subsequent layers are printed effectively, adequate bed leveling is also essential.

### 2.2 Sample preparation

**Fig. 1:**
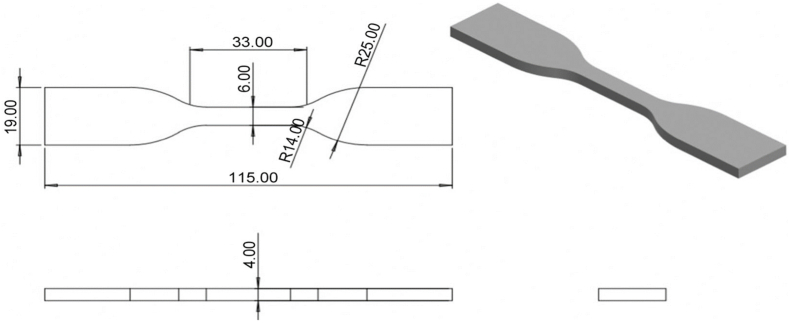
Isometric view of standard ASTM D638 Type IV using Solidwork 2022.

**Fig. 2:**
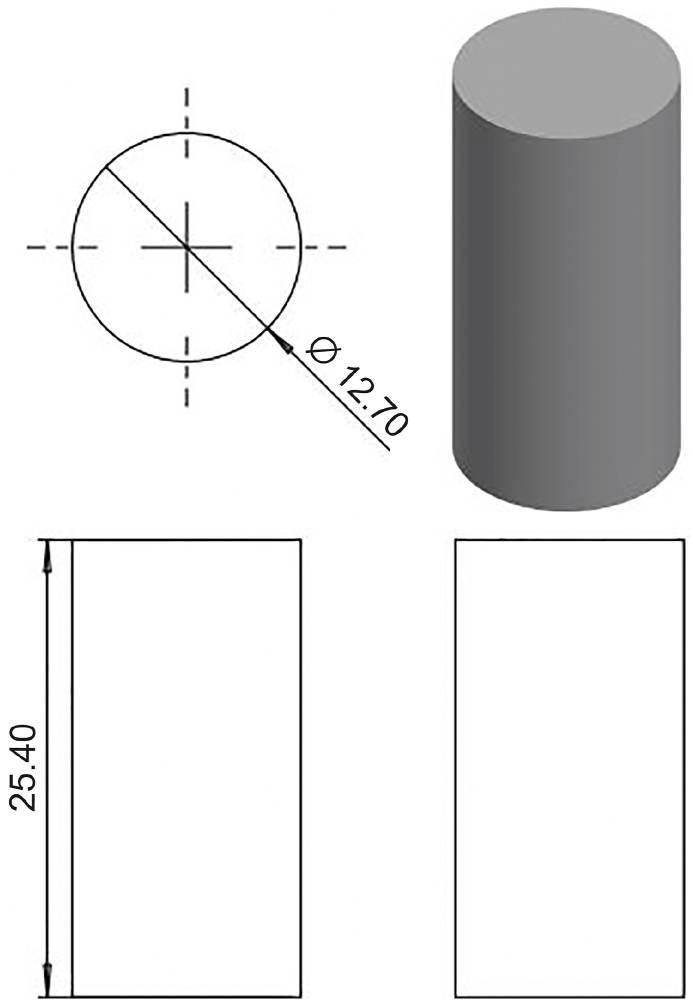
Isometric view of standard ASTM D695-15 using Solidwork 2022.

**Fig. 3:**
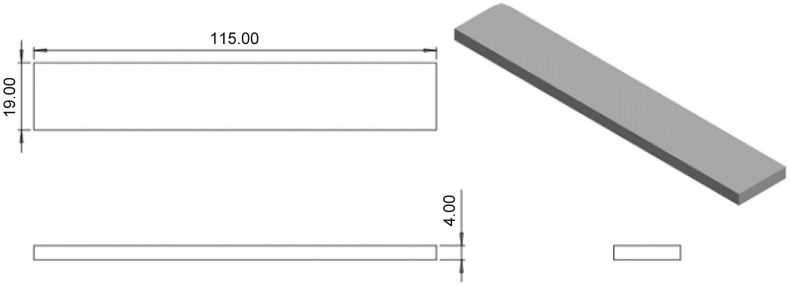
Isometric view of the design for the flexural test using Solidwork 2022.

Mechanical tests were conducted using a universal testing machine. The tensile tests followed the guidelines outlined in ASTM D638 Type IV, the compressive tests adhered to ASTM D695, and the flexural tests were in accordance with ASTM D790. For the tensile tests, the standard design for the mechanical property tests was based on ASTM D638 Type IV. The ASTM D638 Type IV is a dog-bone-shaped specimen ideal for testing very soft polymers and comparing materials of different stiffness. It features a 25-mm gage length with a narrow parallel section with width of 6 mm, wider shoulders of 19 mm, and an overall length of 115 mm. Thickness can vary depending on the material, but typically falls within a range of 3–14 mm. For this study, 3 mm thickness was used. This specific geometry provides a good balance between gripping strength and stress concentration in the gage length, making it suitable for accurate tensile testing of soft materials. Three samples have been fabricated for this test instead of five samples as recommended. The sample can be considered only simple models, and three samples can be considered as optimized resources and balanced between precision and cost. The previous study also used a similar replication sample for the experiment [16]. The developed design is illustrated in Fig. 1. For compressive tests, ASTM D695 is used as the standard design for the mechanical properties’ tests. ASTM D695 uses two common cylindrical specimen sizes: common shape and larger specimen, which is highly versatile. The chosen design had common dimensions of 12.7 mm diameter and 25.4 mm length, making it compact and ideal for measuring various compressive properties such as strength and modulus. The design developed is shown in Fig. 2. Then, for flexural tests, ASTM D790-10 is employed as the standard 3-point flexural test. A cuboid shape with a thickness of 3 mm was used to study the bending properties of the materials. The chosen design featured a 19-mm width and 115-mm length. The design was developed as shown in Fig. 3 to compensate for the testing method.

To establish an effective table for measuring the variations in the samples, the Taguchi technique was applied in this study using Minitab 21.0 (Minitab, USA) software. The temperature and time required for the heat treatment process are two operational variations that were included in Table 2. There are three levels of time and temperature, with ideal values and additional alternatives for each. With the condition of two factors and three levels, nine variations of manipulated factors were developed. For each variation, three samples were prepared to achieve the average value of the data and to ensure consistency throughout the experiment.

**Table 2:** Taguchi table of two factors-three levels.

Run	C1	C2
	Heating Temperature (°C)	Holding Time (min)
1	70	30
2	70	60
3	70	90
4	100	30
5	100	60
6	100	90
7	130	30
8	130	60
9	130	90

**Fig. 4:**
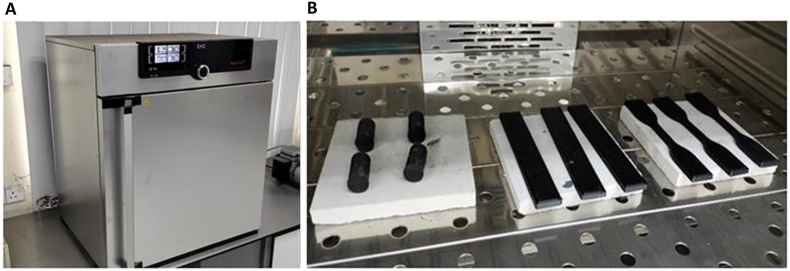
A) Memmert UN30plus B) Specimens being subjected to the annealing treatment.

The heat treatment process, specifying equipment, temperature, and time limits, was conducted using the universal heating oven Memmert UN30plus, as depicted in Fig. 4 A). Renowned for its precision and reliability in materials science and engineering research, this oven offers a slow heating rate and can reach temperatures up to 300 °C, which falls within the parameter range of this study. The Taguchi method identified the holding time and heating temperature as the two crucial variables. The experiment involved three levels of time variations (30 min, 1 h, and 1 h 30 min) and three levels of heating temperature variations (70 °C, 100 °C, and 130 °C). To ensure statistical accuracy, 3 samples were prepared for each condition, totaling 27 samples (9 conditions with 3 samples per condition). Additionally, each sample underwent three mechanical tests: tensile, compressive, and flexural tests, each performed thrice.

This resulted in 81 mechanical tests, as there were 27 samples with 3 tests conducted per sample. The findings of this study are expected to be reliable and insightful because of the application of the Taguchi method, the thorough selection of components and levels, and the comprehensive testing approach, among other considerations. For benchmark and comparison purposes, three nonheat-treated HTPLA samples will be printed, tested, and analyzed.

## 3 Results and discussion

### 3.1 Shrinkage and the dimensional accuracy test

Table 3 shows the outlined findings of the dimensional accuracy and shrinkage analysis for 3D-printed black carbon fiber HTPLA samples subjected to heat treatment. In the tensile test, the Z-axis exhibits a substantial average dimensional accuracy of 10.19 %, which aligns with the manufacturer's information on HTPLA crystallizing at 110 °C to become stiffer. However, the observed shrinkage along the Y-axis (−2.54 %) deviates from the expected 0.5 % x/y shrinkage mentioned by the manufacturer. In flexural testing, the Z-axis experiences substantial shrinkage of 13.78 %, while the X-axis shows slight negative shrinkage, which indicates growth (−0.12 %). These variations could be attributed to the part geometry and setup, as emphasized by the manufacturer. Compressive testing revealed notable shrinkage in both diameter/X–Y-axis (2.45 %) and Z-axis height (negligible at −0.14 %), which may suggest the influence of heat treatment conditions on different aspects of dimensional stability. Comparing these findings with the manufacturer's data, there are consistencies, such as negligible Z-axis change and 0.5 % X/Y shrinkage reported by the manufacturer aligning with certain observed values. However, disparities, particularly in Y-axis shrinkage during tensile testing and variations in flexural testing, indicate considerable material behavior. The manufacturer's guidance on avoiding hot spots and experimenting before baking aligns with the observed deviations, emphasizing the effect of heat treatment. Despite discrepancies, the experimental results provide valuable insights into the complex interplay of heat-treatment conditions and their effects on dimensional accuracy and shrinkage in 3D-printed black carbon fiber HTPLA components. These findings underscored the importance of careful consideration and experimentation in optimizing heat treatment processes for specific geometries and applications.

**Table 3:** Shrinkage and dimensional accuracy of the sample based on the x, y, and z axes.

Test Type	Dimension	Average Shrinkage (%)	Range	Average Dimensional Accuracy (%)	Range
**Tensile**	X-Axis	0.43	−0.12 to 0.77	0.16	−0.39 to 0.49
	Y-Axis	−2.54	−3.87 to 0.07	−1.46	−2.77 to 1.12
	Z-Axis	3.94	−3.21 to 8.38	10.19	−3.5 to 14.33
**Flexural**	X-Axis	−0.12	−0.36 to 0.19	−0.4	−0.64 to −0.09
	Y-Axis	−1.39	−2.2 to −0.84	2.03	−2.84 to −1.47
	Z-Axis	1.46	−4.38 to 4.19	13.78	−8.67 to 16.17
**Compressive**	Diameter/X-Y-Axis	2.45	−1.87 to 3.89	1.62	−0.79 to 2.83
	Height (Z-Axis)	−0.14	−0.69 to 0.87	2.18	1.65 to 3.18

### 3.2 Experimental results for the mechanical properties

The 3D-printed samples of black carbon fiber HTPLA were fabricated using the FDM process for 27 samples for each mechanical test, excluding nine untreated samples. In this experiment, only two parameters were manipulated which are heating temperature and holding time.

The Taguchi technique was employed to determine the most remarkable value that affects the mechanical properties of heat-treated 3D-printed black carbon fiber HTPLA. Fig. 4 B) shows the general appearance of the specimens after being subjected to the annealing treatment. It is possible to observe that there were some geometric alterations in the analyzed specimens. To fully understand the relationships and interactions between the two factors, holding time and heating temperature, further analysis using statistical methods, specifically the Taguchi method, will be applied to each mechanical property. This analysis aimed to examine the correlation between the two variables that may or may not affect the value of tensile strength. All samples were grouped based on variations in factors before conducting mechanical tests.

#### 3.2.1 Effect of heat treatment on tensile strength

In this section, the tensile properties of all printed samples, both with and without heat treatment, were evaluated through mechanical testing, i.e., the tensile test. This analysis aimed to assess the impact of heat treatment on the tensile strength of 3D-printed black carbon fiber HTPLA. The results were presented and discussed using contour plots, analysis of variance (ANOVA), main effect plot. Furthermore, they were observed through scanning electron microscopy (SEM), which provided a visual representation of the influence of heating temperature and holding time.

##### 3.2.1.1 Mechanical properties test

This section discusses the results of the mechanical tests for the tensile samples. The higher tensile strength value indicates the greater the material's resistance to breaking under tension, indicating that the sample is stronger and more durable, while the lower value indicates small resistance to deformation. The experimental results that vary with the two process parameters are shown in Table 4.

**Table 4:** Average tensile strength (MPa) results of each experimental run.

Sample No.	Process Parameters		Results
	Heating Temperature (°C)	Holding Time (min)	Average Tensile Strength (MPa)
1.	70	30	74.5736
2.	70	60	72.5215
3.	70	90	79.5023
4.	100	30	73.0899
5.	100	60	67.8440
6.	100	90	57.6792
7.	130	30	63.1602
8.	130	60	65.5345
9.	130	90	64.2970

The tensile strength of each sample was taken thrice (n = 3) and the average value was then calculated to obtain a more consistent value of the results. The results acquired as shown in Table 4 show that the different combination of process parameters for experimental Run 3 with a combination of 70**°**C of heating temperature and 90 min of holding time has the greatest tensile strength value (79.5 MPa). In comparison, the lowest tensile strength is at 57.68 MPa, obtained from experimental Run 6 with a combination of 70**°**C heating temperature and 90 min holding time, which is lower than that of untreated carbon fiber samples with an average tensile strength of 59.15 MPa.

**Fig. 5:**
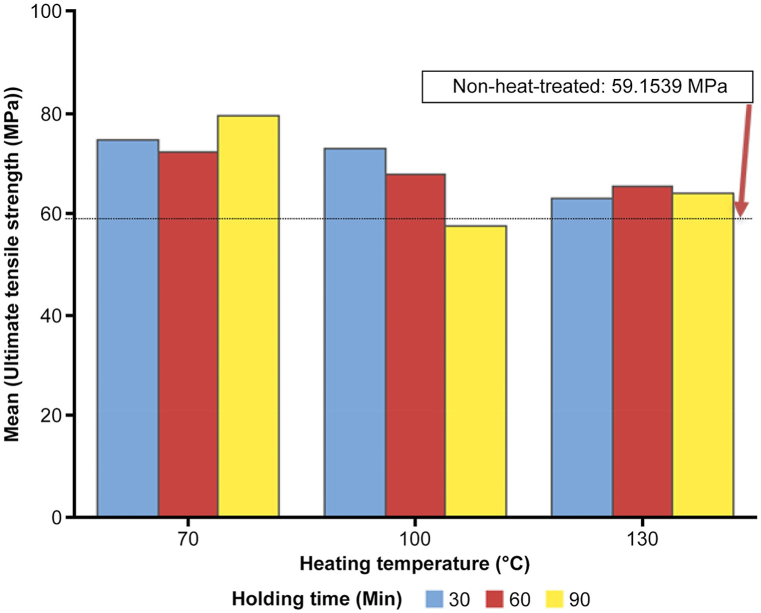
Bar chart of average tensile strength vs. heating temperature and holding time.

The bar chart in Fig. 5 effectively illustrates the tensile strength results for 3D-printed black carbon fiber HTPLA under various heat treatment conditions. The clustered variable is the heating temperature, which is divided into three groups (70 °C, 100 °C, and 130 °C), with each group further distinguished by holding time (30, 60, and 90 min). The reference line at 59.1539 MPa represents the average tensile strength for the nonheat-treated samples, serving as a baseline for comparison. Observations from the chart reveal interesting trends.

At 70 °C, the 90-min holding time stands out, demonstrating a notable increase in the tensile strength compared with the nonheat-treated samples. In contrast, at 100 °C and 130 °C, longer holding times (60 and 90 min) generally result in reduced tensile strength. Visual representation effectively captures the impact of heat treatment on tensile strength, providing insights for optimizing heat treatment parameters to enhance mechanical properties.

**Fig. 6:**
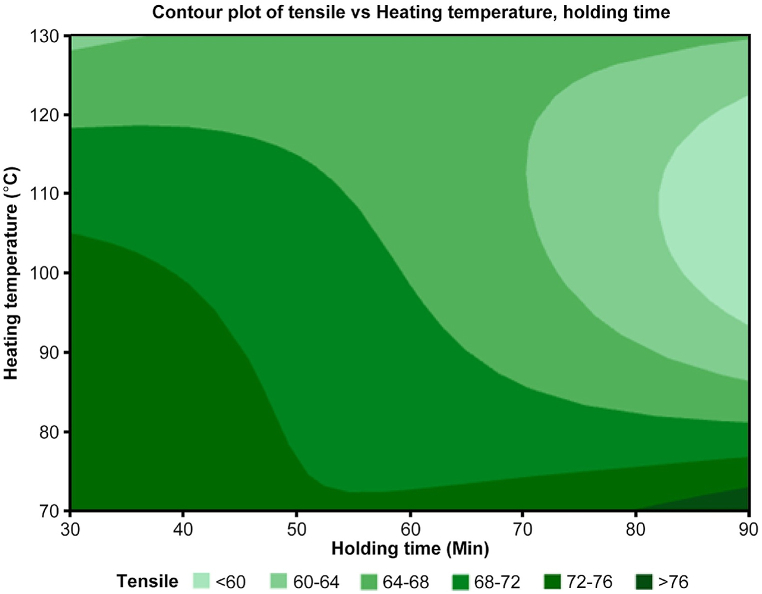
Contour plot of tensile vs. heating temperature and holding time.

Based on the contour plot shown in Fig. 6, the tensile strength of this material only increases until a certain temperature, i.e., 100 °C and then decreases with further heating. This is likely due to the softening of the polymer matrix at higher temperatures. The holding time at each temperature also influences the tensile strength, with longer holding times leading to higher strengths only for the specific heating temperature of 70 °C. This observation is likely due to the increased diffusion of the polymer chains into the carbon fibers, which improves the interfacial bonding between the two materials. This condition can only be met at a certain combination of heating temperature and holding time to achieve minimal damage to the fiber and polymer matrix with optimal diffusion of the polymer chains.

##### 3.2.1.2 ANOVA

Regression analysis is particularly well suited for analyzing the relationship between tensile strength and two factors—heating temperature and holding time. In this context, regression serves as a powerful statistical tool for quantifying and understanding the nature of the influence that varying levels of these factors have on the response variable—tensile strength. This analysis helps identify patterns, trends, and potential correlations, providing valuable insights into how changes in the independent variables impact the dependent variable.

As the significance level for the ANOVA shown in Table 5, a p-value of 0.05 was selected for statistical significance. Based on the findings from Table 4, heating temperature (p = 0.04) was found to have a significant effect on the response variable. However, the regression value shows a p-value of 0.09, which is greater than 0.05. This suggests that there is insufficient evidence to conclude that the independent variables have a significant effect on the dependent variable. Based on the ANOVA analysis, the two factors, heating temperature and holding time, may not significantly impact the tensile strength value.

**Table 5:** Regression analysis of tensile vs. heating temperature and holding time.

Source	DF^a^	Adj SS^b^	Adj MS^c^	F-Value	P-Value
Regression	2	202.78	101.39	3.70	0.090
Heating Temperature	1	188.22	188.22	6.86	0.040
Holding Time	1	14.56	14.56	0.53	0.494
Error	6	164.57	27.43		
Total	8	367.35			

a Degree of freedom.

b Adjusted sum of squares.

c Adjusted mean squares.

**Fig. 7:**
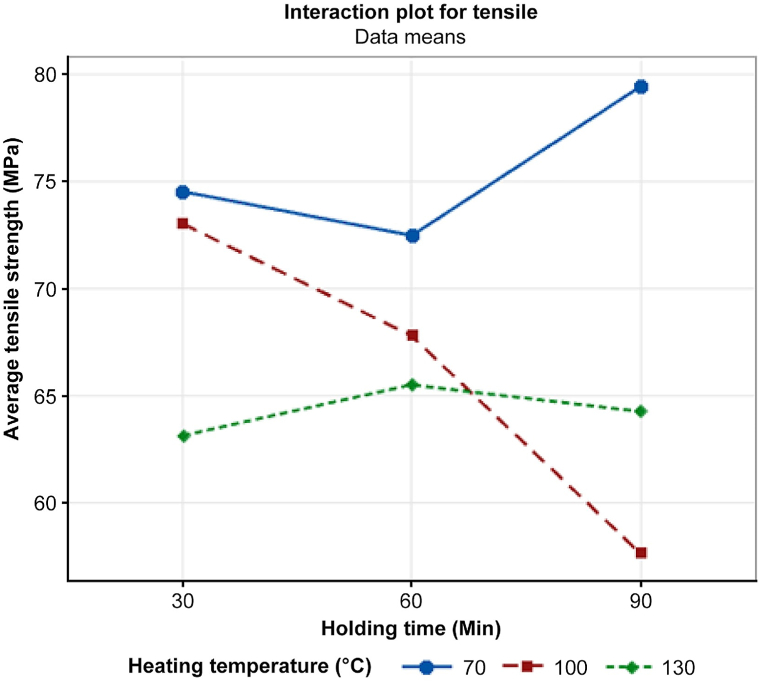
Interaction plot of tensile strength vs. heating temperature and holding time.

Even though the ANOVA analysis suggests that the factors may not have a significant impact, further investigation was carried out by examining the pattern observed in the interaction plot of the tensile test, as depicted in Fig. 7. It was observed that heating temperature only affects the tensile strength at low temperatures. This finding aligns with previous research by Mariam Shbanah et al. [17], indicating that the optimal heating temperature for PLA polymers falls within the range of 65 °C–80 °C, with variations in holding time.

**Fig. 8:**
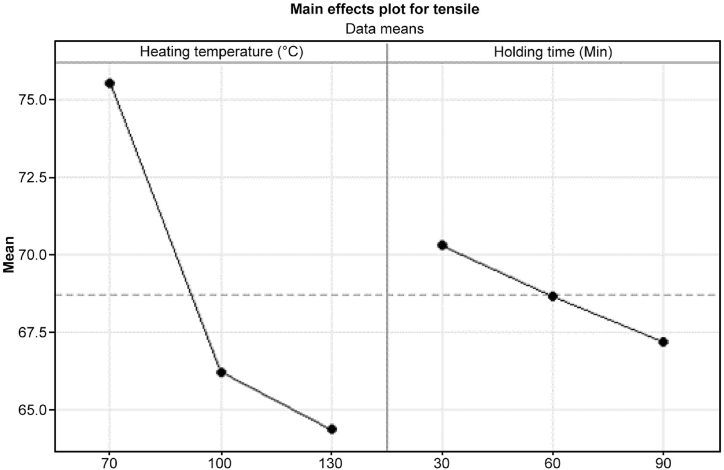
Main effects plot of tensile strength with heating temperature and holding time.

For further analysis, the main effect was plotted as shown in Fig. 8, showing the impact of heating temperature and holding time on tensile strength. Notably, optimal performance, with the highest mean tensile strength of 75.53 MPa, was observed at 70 °C. However, a decreasing trend in tensile strength was noted with rising temperatures. Additionally, holding time showed a diminishing effect on tensile strength, with the highest mean value recorded at 30 min, reaching 70.27 MPa. These findings can be further explored through microstructure analysis. The sample with a combination of 70 °C and 30 min was specifically examined. It was evident that high-temperature treatments led to considerable deformation in the treated specimens, particularly at 95 °C [17].

##### 3.2.1.3 Microstructural analysis

**Fig. 9:**
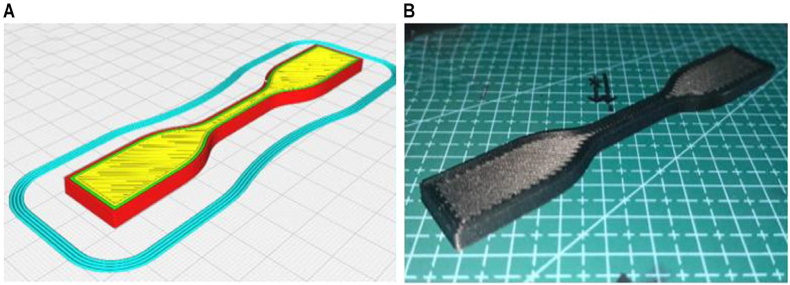
A) 3D model in the CURA Slicer and B) finished product of 3D printing.

**Fig. 10:**
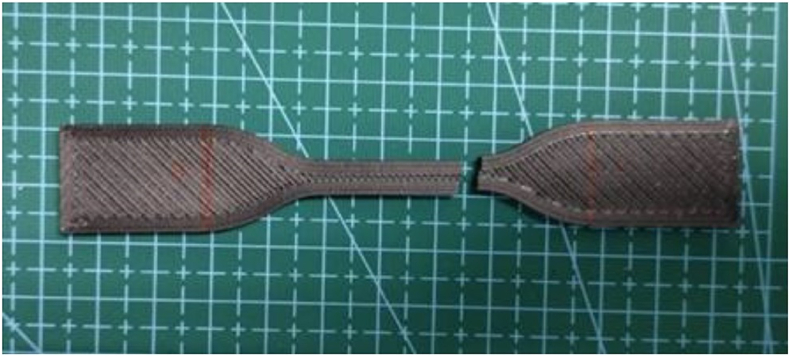
Fractured location of the nonheat-treated sample.

After the tensile test was conducted, the specimen surfaces were examined via visual SEM. The microscopy test showed differences as a function of the heating temperature and the holding time affecting the fusion and absence of air gaps and voids in the interlayer of the printed samples. Fig. 9 shows A) the design of the dog-bone-shaped specimen in the CURA Slicer and B) after the 3D-printing process, following the guidelines set by ASTM D638 for the tensile test. This specific geometry is essential for evaluating the material's resistance to tension, providing valuable insights into tensile strength and related mechanical properties. The CURA Slicer depiction emphasizes the meticulous layering and infill patterns integral to accurate and consistent tensile testing. The selected samples were observed using SEM at the fractured surface, as shown in Fig. 10.

**Fig. 11:**
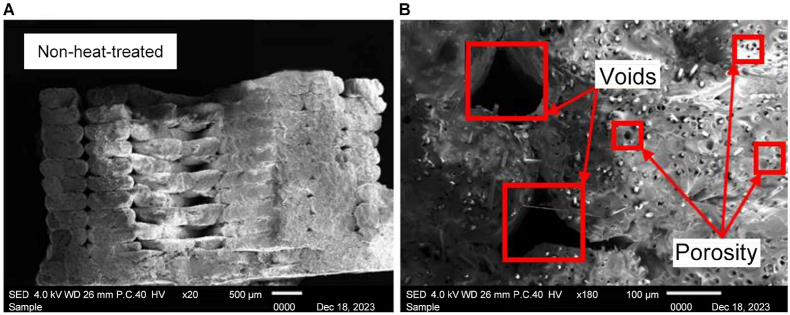
Scanning electron microscopy images of the nonheat-treated sample at A) × 20 and B) × 180 magnification.

In Fig. 11, the ruptured surface of the nonheat-treated sample is depicted. Fig. 11 A) shows clear voids are observed, emphasizing the lack of fusion between the layers. Upon closer inspection at × 180 magnification, distinct voids and porosity become more pronounced as shown in Fig. 11 B). The SEM analysis revealed the presence of air gaps between the fibers and the PLA base, underscoring the impact of the absence of heat treatment on the microstructural integrity of the sample.

**Fig. 12:**
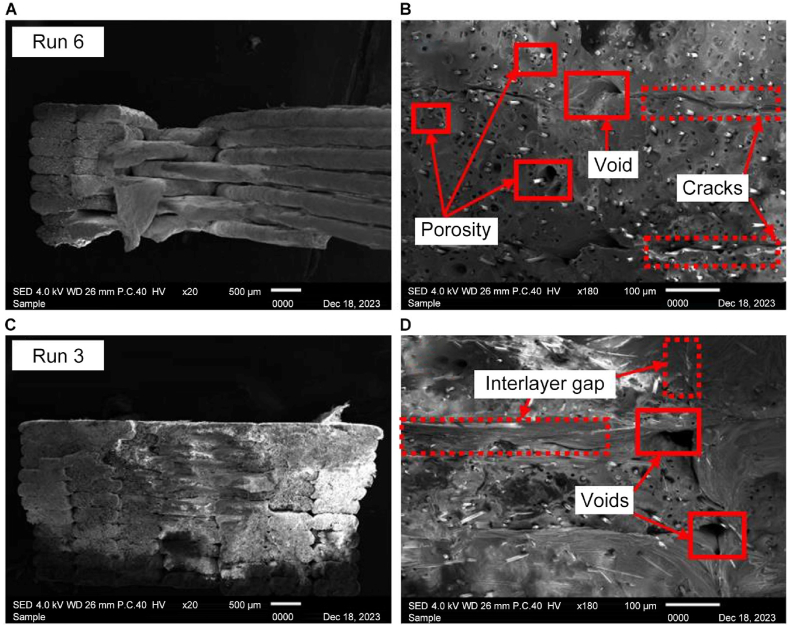
Scanning electron microscopy (SEM) images of Run 6 sample at A) × 20 and B) × 180 magnification. SEM images of the Run 3 sample at C) × 20 and D) × 180 magnification.

Owing to the nature of the heat treatment process, sample 6 shown in Fig. 12 A), with a combination of 100 °C of heating temperature and 90 min of holding time, shows the gaps healed by an increase in temperature but revealed that the properties of the material changed to be more brittle as the crack interlayer is shown in Fig. 12 B) without complete fusion between the layers. There are still evident gaps between the layers. The SEM analysis highlights the positive effect of heat treatment in mitigating voids and enhancing interlayer adhesion. Fig. 12 C) illustrates the rupture surface of Run 3 with the highest tensile strength after heat treatment. With a combination of 70 °C at 90 min holding time, shown at Fig. 12 C) × 20 magnification, a neat arrangement and strong adhesion between layers are apparent. Fig. 12 D) × 180 magnification reveals that while the voids are considerably healed, there are traces of voids remaining. This sample has the characteristic of a ductile material, which may lead to the highest strength. The microstructural analysis further indicates good adhesion between layers, absence of porosity, and a homogeneous crystalline structure in the PLA base, demonstrating the efficacy of the chosen heat treatment parameters.

**Fig. 13:**
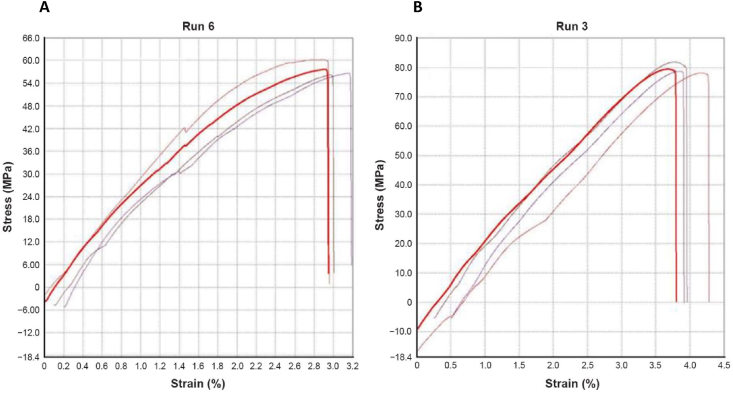
A) Stress–strain (MPa) curves of samples for Run 6 B) Stress–strain (MPa) curves of samples for Run 3.

Fig. 13 A) and B) compare the critical change in the curves of the stress–strain diagram of both samples from Runs 6 and 3. The curves show the highest tensile strength yield by Run 6 only peak at 60 MPa with an average of only 57.68 MPa. Meanwhile, the stress–strain diagram yield by Run 3 shows the highest tensile strength, which peaks at around 84.00 MPa with an average of 79.50 MPa, showing a positive effect with lower heating temperature.

**Fig. 14:**
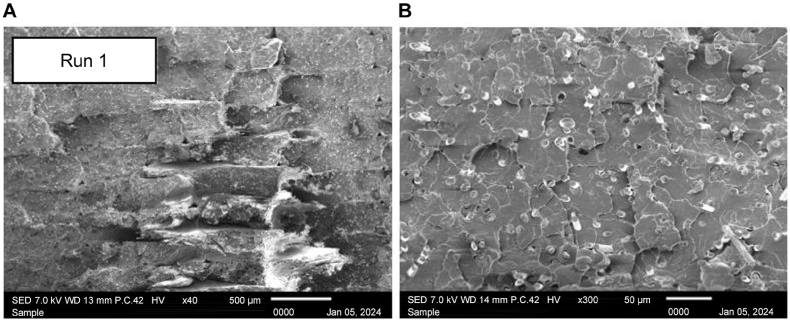
Scanning electron microscopy images of Run 1 sample at A) × 40 and B) × 300 magnification.

Then, analysis of samples of Run 1 shown in Fig. 14 A) at ×40 magnification and Fig. 14 B), ×300 magnification representing the optimum combination for the highest tensile strength with good fusion between layers and minimal seven voids, provides crucial insights into the effectiveness of the chosen parameters—the combination of 70 °C heating temperature and 30 min holding temperature. The observed superior tensile strength indicates that this selected combination of heating temperature and holding time has a positive impact on the overall structural integrity of the 3D-printed black carbon fiber HTPLA. The presence of minimal voids indicates improved layer adhesion and a more homogenous structure, contributing to the enhanced mechanical performance. The optimization of these parameters may lead to increased tensile strength, highlighting the effectiveness of the heat treatment process in achieving a well-fused and void-free microstructure. This observation holds true even though, based on the statistical analysis, these factors do not appear to have a significant impact on tensile strength.

Overall, the analysis results indicate that the tensile strength of the sample subjected to heat treatment is substantially higher than that of the non-heat-treated sample. While the non-heat-treated sample showed a tensile strength of 59.1539 MPa, Sample 1 achieved a significant improvement, boasting an average tensile strength of 74.5736 MPa.

#### 3.2.2 Effect of heat treatment on compressive strength

This section comprehensively examines the influence of heat treatment on the compressive strength of 3D-printed black carbon fiber HTPLA specimens. The mechanical test results quantify the impact on compressive strength, ANOVA discerns statistically significant variations, and microstructure images provide visual insights into the microstructural changes. Together, these findings offer a comprehensive understanding of how heat treatment affects the material's compressive strength.

##### 3.2.2.1 Mechanical properties test

**Fig. 15:**
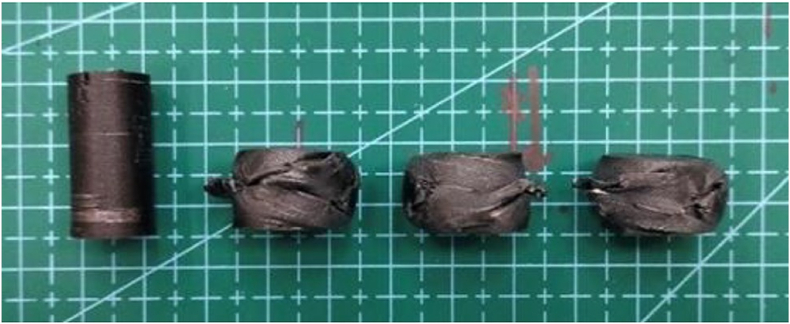
Compressive sample before and after the test.

This section details the mechanical testing of all compressive samples using the SHIMADZU Autograph AGSX Universal Tensile machine (250 kN) at 15 mm/min until half the sample height is reached. The experimental results vary with the two process parameters which are heating temperature and holding time. Additionally. Fig. 15 illustrates the compressive values before and after the test, visualizing to highlight the dimensional changes and reductions for direct comparison of the sample's structural transformation under applied force. It is important to note that the compressive test was carried out until half of the sample's height, equivalent to a gap of 12.70 mm, was reached before the test was concluded. This specific testing protocol ensures a standardized and controlled assessment of the compressive strength properties of the material.

The data in Table 6 show the results of the combination of heating temperature and holding time for compressive strength. For instance, sample 7, treated at 130 °C for 30 min exhibited a compressive strength of 71.73 MPa, whereas those treated at 130 °C for 60 min demonstrated a further increase to 78.95 MPa before it decreased again at 90 min with 70.84 MPa. A decrease in compressive strength is observed with longer holding times for the highest temperature.

**Table 6:** Average compressive strength (MPa) results of each experimental run.

Sample No.	Process Parameters		Results
	Heating Temperature (°C)	Holding Time (min)	Average Compressive Strength (MPa)
1.	70	30	74.6346
2.	70	60	69.4598
3.	70	90	73.2513
4.	100	30	71.1264
5.	100	60	75.8657
6.	100	90	77.4881
7.	130	30	71.7319
8.	130	60	78.9480
9.	130	90	70.8413

**Fig. 16:**
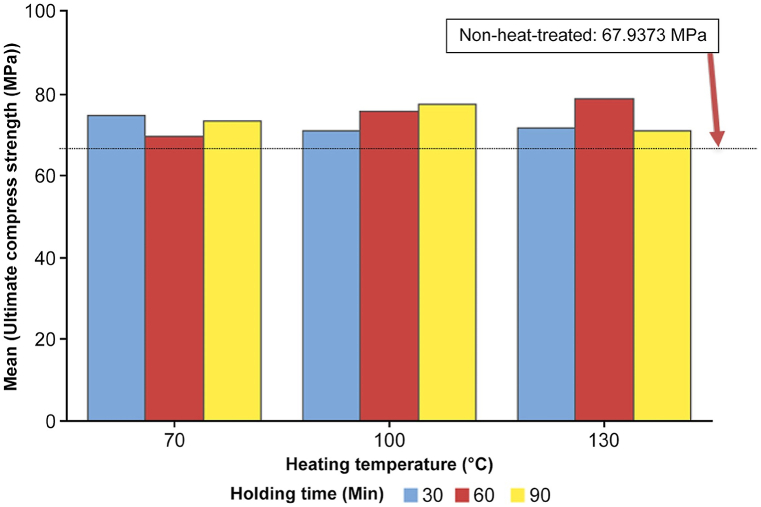
Bar chart of average compressive strength vs. heating temperature and holding time.

The bar chart shown in Fig. 16 provides a visual representation of the compressive strength results for 3D-printed black carbon fiber HTPLA under various heat treatment conditions. The clustered variable is the heating temperature, which is categorized into three groups (70 °C, 100 °C, and 130 °C), with each group further differentiated by holding time (30, 60, and 90 min). The reference line at 67.9373 MPa represents the average compressive strength for the nonheat-treated samples, serving as a baseline for comparison. Analysis of the chart reveals interesting trends. At 70 °C, the 60-min holding time demonstrates a dip in compressive strength compared with nonheat-treated samples, whereas other holding times show a slight increase. At 100 °C, longer holding times (60 and 90 min) generally result in higher compressive strength. However, at 130 °C, the trend suggests that the 60-min holding time yields the highest compressive strength. This visual representation effectively highlights the intricate effects of heat treatment on compressive strength, offering insights for refining the heat treatment process to optimize mechanical properties.

**Fig. 17:**
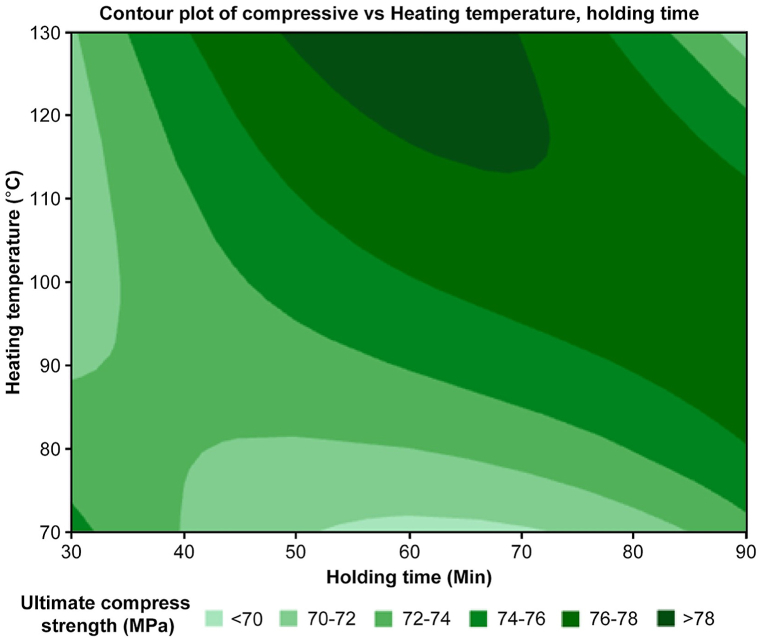
Contour plot of compressive vs. heating temperature and holding time.

To study the effect of heating temperature and holding time on the compressive properties of the optimal combination. The contour plot shown in Fig. 17 depicts the average compressive strength in the form of concentration shades. The most concentrated shades are around 130 °C and 60 min, proposing that this combination achieves the highest compressive strength. Furthermore, the contour lines become slightly elongated toward the holding time, resulting in a decrease in compressive strength at 90 and 30 min, showing that long exposure to heat might lead to a negative effect on mechanical properties. While the highest strength is achieved at 130 °C and 60 min, the optimal combination for practical applications will be analyzed and discussed through ANOVA in the next section.

##### 3.2.2.2 ANOVA

**Fig. 18:**
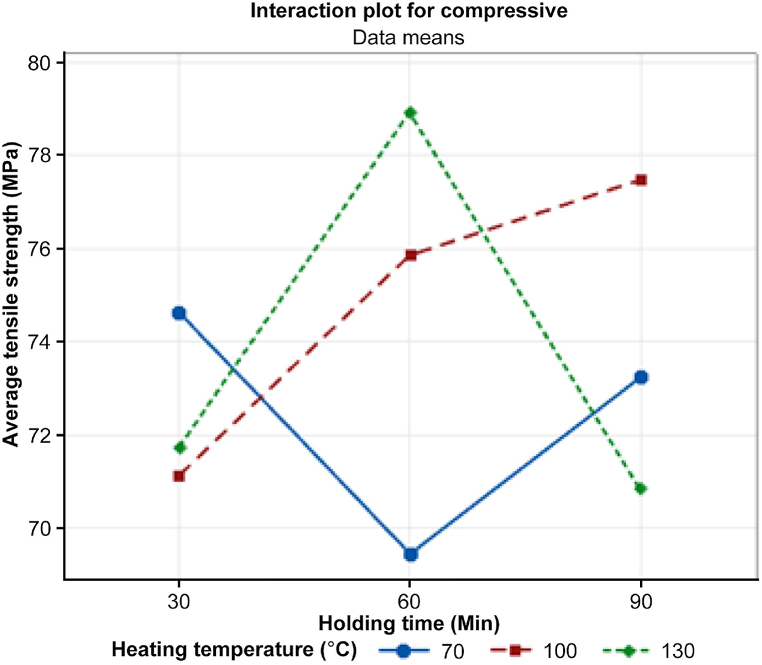
Interaction plot for Compressive strength with heating temperature and holding time.

Although the ANOVA table in Table 7 shows no significant difference between the means of the two groups (p > 0.05), more insights can be gained by examining the interaction plot of the tensile test displayed in Fig. 18.

**Table 7:** Regression analysis: compressive versus heating temperature, holding time.

Source	DF^a^	Adj SS^b^	Adj MS^c^	F-Value	P-Value
Regression	2	5.691	2.845	0.22	0.811
Heating Temperature	1	2.906	2.906	0.22	0.654
Holding Time	1	2.785	2.785	0.21	0.661
Error	6	78.613	13.102		
Total	8	84.304			

a Degree of freedom.

b Adjusted sum of squares.

c Adjusted mean squares.

Fig. 18 shows that the mean of the heating temperature was slightly lower than the mean of the other groups. This suggests that there may be a substantial difference between the means of the groups depending on the experimental size, even if it is not statistically significant. The interaction plots in Fig. 18 suggest a potential combination of heat treatment temperature and holding time for maximizing the compressive strength of 3D-printed carbon fiber HTPLA. Based on the results, the optimal heat treatment temperature appears to be 130 °C, and the optimal holding time appears to be 30 min to achieve the highest compressive strength.

**Fig. 19:**
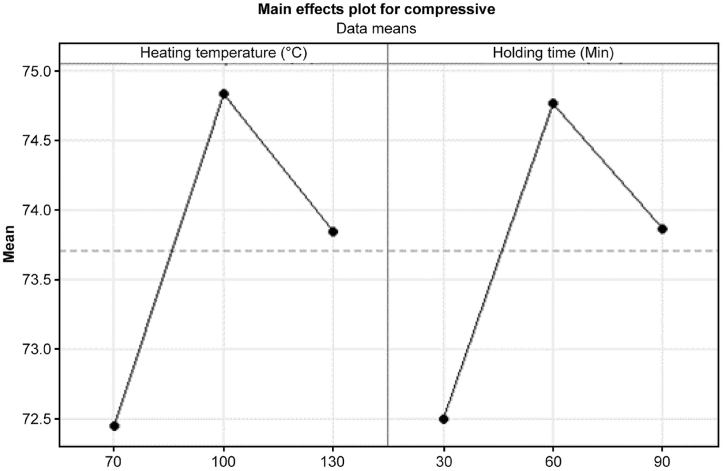
Main effects plot of compressive strength with heating temperature and holding time.

Furthermore, this study aimed to investigate the impact of heating temperature and holding time on the compressive strength of 3D-printed black carbon fiber HTPLA. The main effect plot displayed in Fig. 19 revealed a positive correlation between heating temperature and strength, indicating enhanced particle bonding at optimal temperatures. Conversely, a negative correlation with holding time implies potential material degradation with prolonged exposure.

Despite the statistical analysis suggesting the insignificance of these two factors, this study revealed that the maximum strength is somehow associated with a specific temperature and holding time. The main effects plot for compressive strength is particularly noteworthy, which highlights significant enhancements in the mean value for the sample subjected to 100 °C heating temperature and 60 min of holding time. In brief, these findings offer valuable insights for process optimization. Further analysis involved observing the structural analysis of the printed.

##### 3.2.2.3 Microstructural analysis

**Fig. 20:**
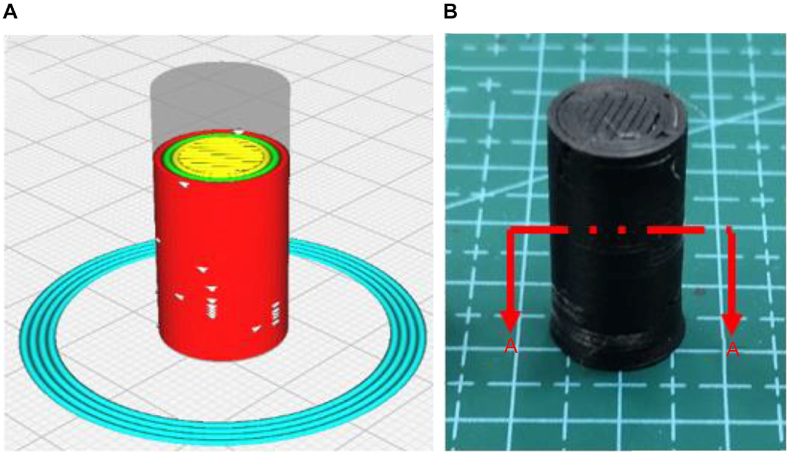
A) 3D digital model in the CURA Slicer, and B) finish product of 3D printing.

The microstructural analysis provides valuable insights into the characteristics of materials, allowing for a detailed examination of the impact of heat treatment on compressive strength. Fig. 20 A) illustrates the design of the cylindrical specimen in the CURA Slicer and Fig. 20 B) after the 3D printing process, which is crucial for influencing the structural integrity and mechanical behavior of the final printed sample. This compressive cylindrical specimen adheres to the standards outlined in ASTM D695. CURA Slicer, a widely used 3D-printing software, translates the 3D digital model into precise instructions for the 3D printer, ensuring accurate replication of the intended geometry.

**Fig. 21:**
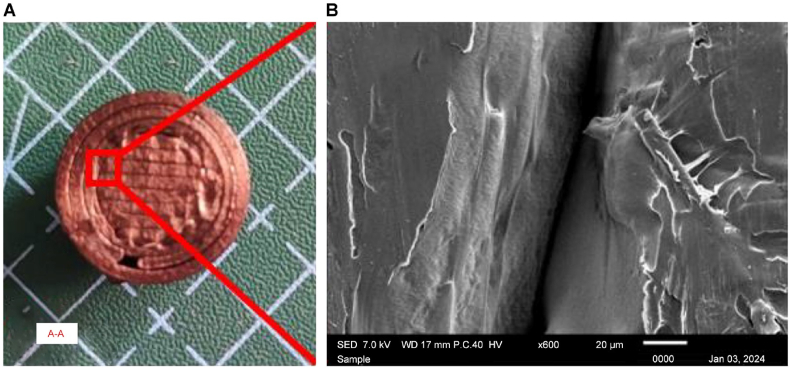
A) A–A cross-section of the 3D printed sample and B) magnification at ×600 of the gap at the cross-sectional area.

To further elucidate the microstructural changes induced by heat treatment on compressive strength, an in-depth analysis will be conducted on the cross-sectional view along the A–A axis shown in Fig. 21 A) and Fig. 21 B). This cross-sectional examination using SEM will allow for a detailed investigation of internal features, such as layer adhesion, voids, and potential defects within the printed samples. The A–A cross-section serves as a critical plane for assessing how heat treatment influences the material's internal architecture, providing valuable information on the factors contributing to changes in compressive strength at the microscale.

**Fig. 22:**
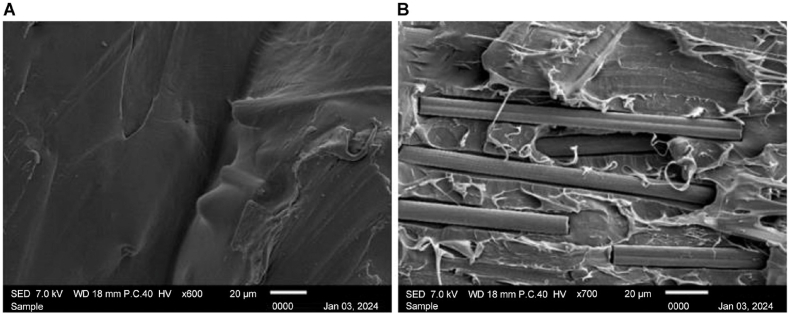
Scanning electron microscopy images of nonheat-treated samples at A) × 600 and B) × 700 magnification.

The image, captured at × 600 magnification, is shown in Fig. 22 A. The nonheat-treated sample serves as a baseline reference. The gaps between the filaments are visible, indicating the initial state of the printed material without any thermal postprocessing. The lack of fusion at adjacent layers revealed the depth of the gaps between layers. The surface shown in Fig. 22 B) at × 700 magnification exhibits some irregularities, and the absence of heat treatment results in less fusion between the fiber and the PLA base. This serves as a benchmark for comparing structural features with their heat-treated counterparts.

**Fig. 23:**
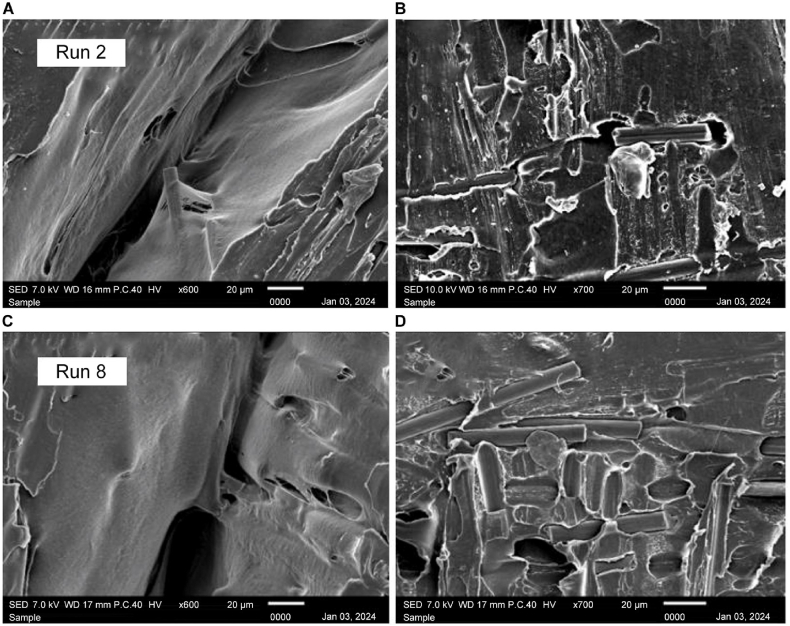
Scanning electron microscopy (SEM) images of the Run 2 sample at A) × 600 and B) × 700 magnification; SEM images of the Run 8 sample at C) × 600 and D) × 700 magnification.

Fig. 23 A) shows the sample with the lowest compressive strength with manipulated parameters of 70 °C heating temperature at 60 min of holding time. Notable characteristics include voids, poor layer adhesion Fig. 23 A), and visible signs of filament separation Fig. 23 B). The structural weaknesses seen in this figure contribute to the reduced compressive strength of the sample. Identifying these features helps to pinpoint the factors that negatively impact material integrity.

At × 700 magnification, Fig. 23 C) provides an intricate view of a sample of Run 8, which was treated at 130 °C with 60 min of holding time. The surface reveals details such as well-fused fibers, minimal voids, and a uniform surface, suggesting optimal layer adhesion and overall structural integrity. The magnification at × 600 shown in Fig. 23 D) also shows that the gaps in the interlayer are shallow, proving that the heating treatment assists in optimizing the microstructure of the sample.

**Fig. 24:**
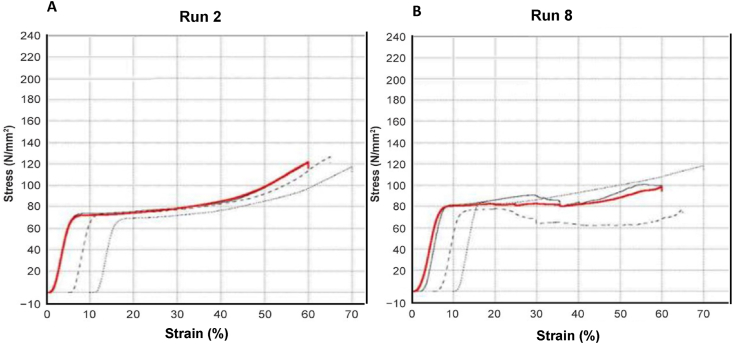
A) Stress–strain (MPa) curves of sample Run 2 B) Stress–strain (MPa) curves of sample Run 8.

Fig. 24 A) and B) compare the critical change in the stress-strain diagram of both samples from Runs 2 and 8. The curves indicate that the highest tensile strength was yielded by Run 6, peaking at around 70 MPa with an average of 69.4598 MPa. In contrast, the stress-strain diagram yielded by Run 8 shows the highest tensile strength, peaking at around 80.00 MPa with an average of 78.95 MPa. This demonstrates a positive effect in increasing the heating temperature, albeit only at certain temperatures and holding times, as indicated by the main effect plot. Therefore, the sample corresponding to this parameter combination was further examined.

**Fig. 25:**
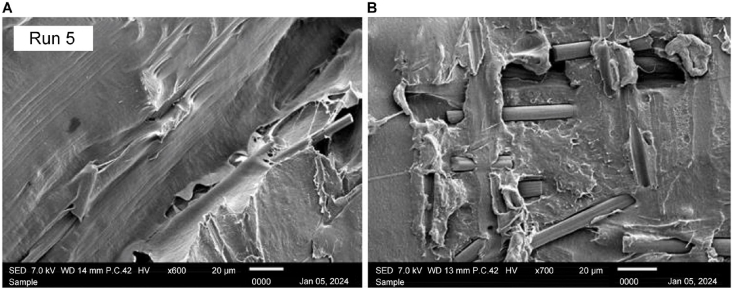
Scanning electron microscopy images of the Run 5 sample at A) × 600 and B) × 700 magnification.

Based on the main effect plot, it is suggested that the sample from Run 5 offers superior compressive strength. Fig. 25 represents the sample that has resulted in an optimal compressive strength value. Fig. 25 A) × 700 magnification, the surface is examined for characteristics such as complete fiber-PLA fusion, minimal voids, and a well-defined microstructure. Fig. 25 B) also provides insights into the shallowness of the gaps between layers, helping to identify the ideal conditions for achieving an optimum compressive strength of 75.8657 MPa in the compressive printed samples.

The main finding reveals that the heat treatment process does influence the compressive strength of 3D-printed black carbon fiber HTPLA. A previous study has also demonstrated a significant impact on improving the mechanical properties post-heat treatment [18]. This study further analyses the effect of two parameters, which are heating temperature and holding time, to assess their impact on compressive strength. The ANOVA results indicate no significant differences between these two parameters, prompting further investigation through interaction plot analysis and examination of the microstructure of the printed parts. It can be inferred that these two combinations may somehow affect compressive strength and could be considered for enhancing the heat treatment process.

#### 3.2.3 Effect of heat treatment on flexural strength

This section examines the impact of heat treatment on flexural strength and investigates whether altering the heat treatment conditions can influence the flexural strength of 3D-printed black carbon fiber HTPLA specimens.

##### 3.2.3.1 Mechanical properties test

**Fig. 26:**
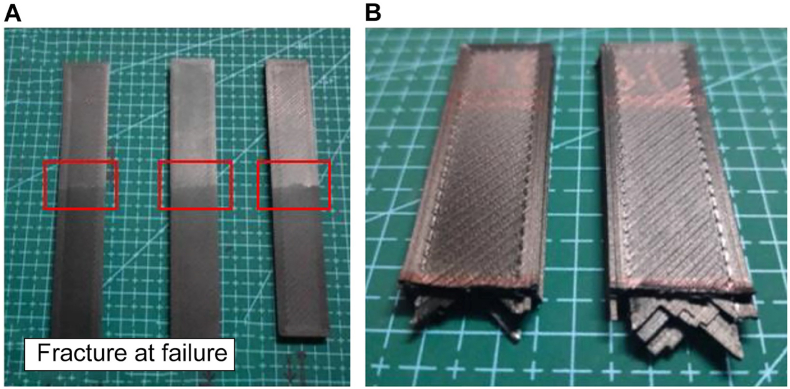
Run 3 A) samples after the flexural test and B) texture of the sample after being broken into half.

To explore the influence of heat treatment on flexural strength, experimental runs were conducted with varying heating temperatures and holding times. Flexural tests were performed using a SHIMADZU Autograph AGSX Universal Tensile machine (5 kN), measuring the resistance of the material to bending. Fig. 26 A) shows the crack characteristics on the flexural samples and B) the texture of the materials after breaking into half.

**Fig. 27:**
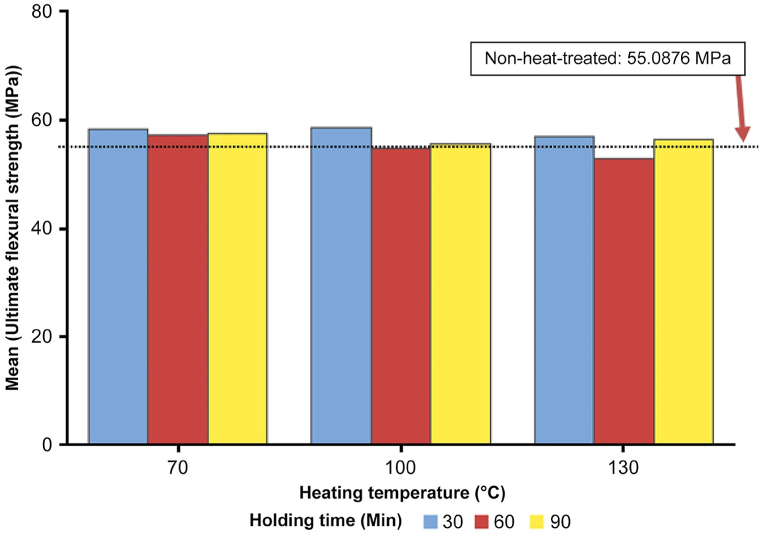
Bar chart of average flexural strength vs. heating temperature and holding time.

The initial analysis aims to evaluate the impact of heat-treated samples compared to non-heat-treated samples. The bar chart presented in Fig. 27 clearly visualizes the flexural strength results for 3D-printed black carbon fiber HTPLA under various heat treatment conditions. The clustered variable is the heating temperature, which is categorized into three groups (70 °C, 100 °C, and 130 °C), with each group further differentiated by holding time (30, 60, and 90 min). The reference line at 55.0876 MPa represents the average flexural strength for the nonheat-treated samples, serving as a baseline for comparison. Observations indicate variations in the flexural strength based on different heat treatment parameters. Notably, at 70 °C, the 30-min holding time showed a slightly increased flexural strength compared with nonheat-treated samples, whereas longer holding times exhibited comparable or slightly lower strength. The chart effectively captures the nuanced effects of heat treatment on flexural strength, offering valuable insights for optimizing the heat treatment process for enhanced mechanical properties [18].

The obtained results are summarized in Table 8. Flexural strength was determined for each sample through three tests (n = 3), and the average value was calculated for consistency. Analysis revealed that experimental Run 4, with a heating temperature of 100 °C and a holding time of 30 min, yielded the highest average flexural strength at 58.5873 MPa. Runs 1 and 7 also exhibited notable strength, averaging 58.3180 and 57.0349 MPa, respectively.

**Table 8:** Average Flexural strength (MPa) results for each experimental run.

Sample No.	Process Parameters		Results
	Heating Temperature (°C)	Holding Time (min)	Average Flexural Strength (MPa)
1.	70	30	58.3180
2.	70	60	57.2639
3.	70	90	57.5552
4.	100	30	58.5873
5.	100	60	54.7834
6.	100	90	55.8138
7.	130	30	57.0349
8.	130	60	52.8819
9.	130	90	56.5492

**Fig. 28:**
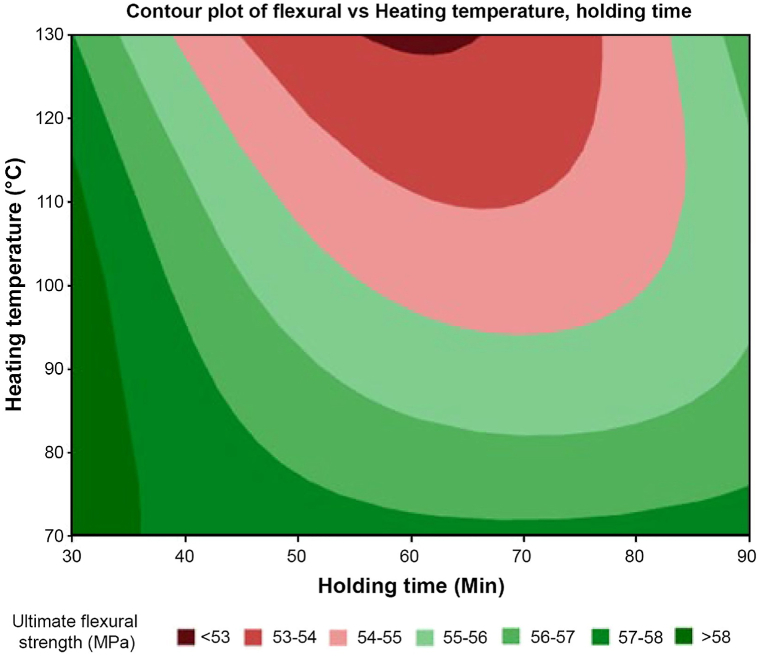
Contour plot of flexural vs. heating temperature and holding time.

The contour plot shown in Fig. 28 depicts the relationship between flexural strength, heating temperature, and holding time. It offers a visual representation of the material's behavior and highlights the heat treatment effect on the 3D-printed samples via the concentration of the shades. Darker green shades signify higher flexural strength, whereas darker red shades indicate lower strength. The plot reveals a general decrease in flexural strength with increasing temperature, reaching its lowest point around 130 °C heating temperature with a holding time of 60 min. Moreover, the holding time at each temperature influences flexural strength, with longer durations yielding higher strengths at lower heating temperatures. Notably, the plot highlights the optimal flexural strength occurring at 70 °C, while an adverse effect is observed with increasing heating temperature.

##### 3.2.3.2 ANOVA

Table 9 suggests that the overall regression model lacks statistical significance, with a p-value of 0.147 exceeding the typical threshold of 0.05. This indicates potential unreliability in predicting flexural strength based on the given data. The small sample size of only nine groups of samples might contribute to this issue. However, the p-value associated with the heating temperature is lower than the other factors, suggesting potential interactions between the investigated factors.

**Table 9:** Regression analysis: flexural vs. heating temperature and holding time.

Source	DF^a^	Adj SS^b^	Adj MS^c^	F-Value	P-Value
Regression	2	10.113	5.057	1.89	0.231
Heating Temperature	1	7.417	7.417	2.78	0.147
Holding Time	1	2.696	2.696	1.01	0.354
Error	6	16.033	2.672		
Total	8	26.147			

a Degree of freedom.

b Adjusted sum of squares.

c Adjusted mean squares.

Further analysis was conducted to assess the potential interaction between the two parameters that affect the tensile strength value. The contour plot shown in Fig. 28 depicts the relationship between flexural strength, heating temperature, and holding time. It offers a visual representation of the material's behavior and highlights the heat treatment effect on the 3D-printed samples via the concentration of the shades. Darker green shades signify higher flexural strength, whereas darker red shades indicate lower strength. The plot reveals a general decline in flexural strength with increasing temperature, reaching its lowest point around 130 °C heating temperature with a holding time of 60 min. In addition, the holding time at each temperature influences flexural strength, with longer durations yielding higher strengths at lower heating temperatures.

**Fig. 29:**
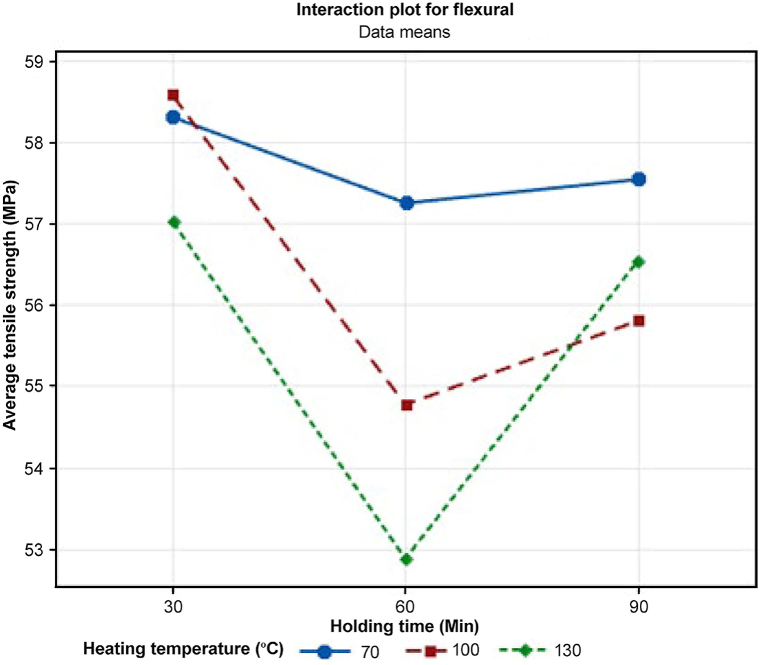
Interaction plot for flexural strength with heating temperature and holding time.

Another analysis was conducted on the interaction plot in Fig. 29. The result was generated from Minitab by ANOVA to emphasize the mean flexural strength of a material at various temperatures and holding times. Each line in the plot represents the mean flexural strength at different temperatures. The lines display a general trend of reductions in strength, suggesting that the effect of holding time on flexural strength may be dependent on the temperature. At 70 °C, the mean flexural strength increased slightly with holding time, from 57.2 to 58.3 MPa. At 100 °C, the mean flexural strength remains relatively constant between 56.9 and 57.1 MPa. At 130 °C, the mean flexural strength decreased slightly with holding time, from 57.0 to 56.5 MPa.

**Fig. 30:**
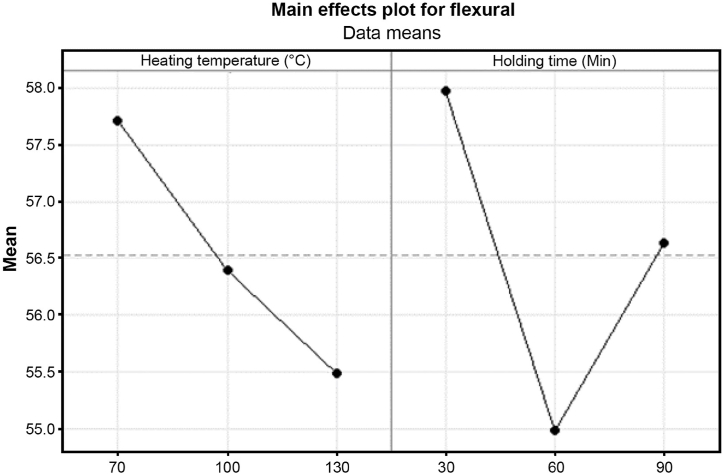
Main effects plot for flexural strength with heating temperature and holding time.

The interaction plot in Fig. 30 indicates a general trend of lower flexural strength with higher temperatures. The heating temperature shows a slightly positive effect at 70 °C with a mean value of 57.71 MPa of flexural strength and decreases with higher temperature, such as at 100 °C with a mean value of 56.38 MPa and at 130 °C with a mean value of 55.49 MPa. Overall, the samples exhibit decreased flexural strength with increasing heat treatment temperature. Then, holding time shows a unique trend where the highest mean value at 30 min is 57.98 MPa and decreases to 54.97 MPa at 60 min before a slight increase at 90 min with a mean value of 56.63 MPa. This might be due to potential damage to carbon fibers or softening of the polymer matrix during the heat treatment process, both contributing to reduced material strength.

##### 3.2.3.3 Microstructural analysis

**Fig. 31:**
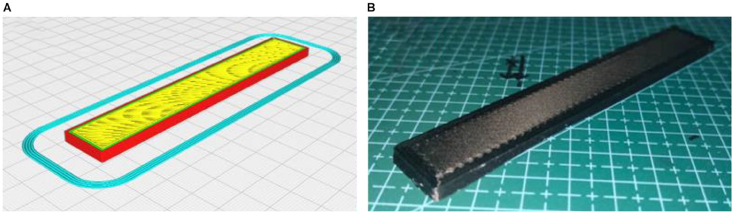
A) 3D model in the CURA Slicer, and B) finish product of 3D printing.

Additional analysis was conducted to investigate the microstructure of the selected samples. This was done to understand and analyze how HTPLA behaves when exposed to various temperatures and its impact on the filament and fusion interlayer of the material. Fig. 31A illustrates the design of the cuboid-shaped specimen in the CURA Slicer, while Fig. 31B shows its appearance after the 3D-printing process. The study was performed according to ASTM D790 for the flexural test. This figure highlights the importance of the cuboid's dimensions and layering pattern in capturing the material's response to heat treatment, which correlates with each remarkable flexural strength value. The design is critical for assessing flexural strength and modulus, providing valuable information about the material's behavior under flexural stress. The role of the CURA Slicer is evident in ensuring the accurate replication of the intended geometry for reliable flexural test outcomes.

**Fig. 32:**
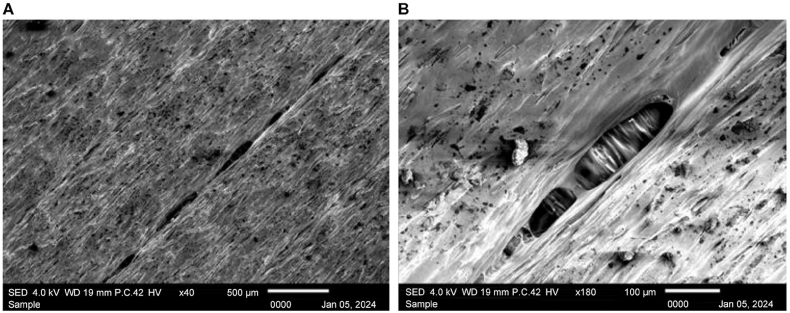
Scanning electron microscopy images of the nonheat-treated sample at a) × 40 and b) × 180 magnification.

First, Fig. 32 A), corresponding to the nonheat-treated sample at × 40 magnification, reveals evident gaps between layers, indicating potential weaknesses in layer adhesion. Upon closer inspection in Fig. 32 B) × 180 magnification, distinct holes and gaps are observed, exposing the layers underneath. These findings demonstrate the importance of heat treatment in addressing structural deficiencies, interlayer fusion, and enhancing the overall integrity of the material.

**Fig. 33:**
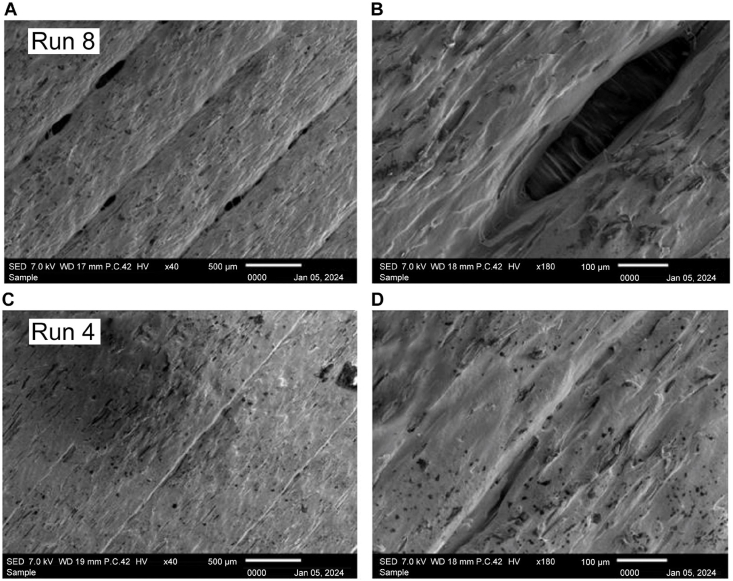
Scanning electron microscopy (SEM) images of Run 8 sample at A) × 40 and B) × 180 magnification. SEM images of Run 4 sample at C) × 40 and D) × 180 magnification.

Next, Fig. 33 shows the sample with the lowest ultimate tensile strength after treatment, which reveals, in Fig. 33 A) × 40 magnification, a conspicuous lack of adhesion between layers; Fig. 33 B) × 180 magnification, further highlights that despite exposure to high temperatures, the voids in the structure remain unhealed. Possible reasons for this suboptimal outcome include overexposure to heat, which leads to shrinking of the layer wideness, emphasizing the sensitivity of the 3D-printed material to the postprocessing conditions. Conversely, Fig. 33 C) and Fig. 33 D) show the sample with the highest ultimate flexural strength after treatment, which at Figure C) × 40 magnification exhibits a remarkably neat arrangement and strong adhesion between layers. Figure D) × 180 magnification, also validates these observations by indicating complete healing of voids, impeccable adhesion, absence of porosity, and lack of discernible gaps. This result proves the efficacy of specific heat treatment conditions in optimizing the microstructure of the material for enhanced mechanical properties.

**Fig. 34:**
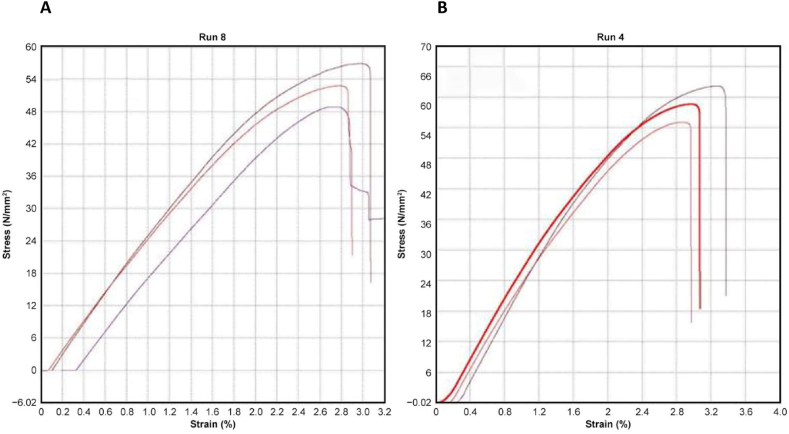
A) Stress–strain (MPa) curves of sample Run 8 B) Stress–strain (MPa) curves of sample Run 4.

Fig. 34 A) and B) compare the critical change in the curves of the stress–strain diagram of both samples from Runs 8 and 4. The curves show the highest tensile strength yield by Run 8 only peak at around 56 MPa with an average of only 52.88 MPa. Meanwhile, the stress–strain diagram yield by Run 4 shows the highest tensile strength, which peaks at around 62.00 MPa with an average of 58.59 MPa, showing a positive effect in maintaining the heating temperature at low temperature with minimum holding time.

**Fig. 35:**
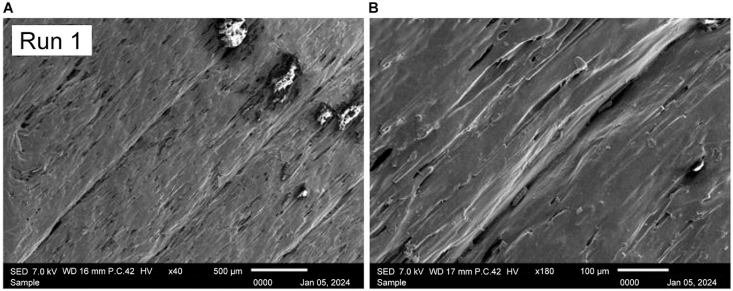
SEM images of Run 1 sample at A) × 40 and B) × 180 magnification.

Lastly, Fig. 35 representing the sample with the best combination of heat treatment parameters reveals, at Fig. 35 A) × 40 magnification, an exceptionally neat and homogenous structure. The SEM image shown at Fig. 35 B) with × 180 magnification further confirms the similarity of this sample's characteristics to those of the highest ultimate tensile strength sample, reinforcing the importance of the identified combination of temperature and holding time in achieving optimal microstructural outcomes.

The significant finding is that heat treatment influences the flexural strength of 3D-printed black carbon fiber HTPLA [19]. Despite the lack of statistical significance between the two parameters in the heat treatment process, namely heating temperature and holding time, as indicated by ANOVA, further investigation has been conducted to identify interaction and observe the microstructure of the samples. Based on the analysis, it is suggested that these two parameters (heating temperature and holding time) might have an effect on the overall performance and could be considered for enhancing the mechanical flexural strength of the printed parts.

## 4 Conclusions

In conclusion, this research aimed to examine the impact of heat treatment on 3D-printed black carbon fiber HTPLA. The study focused on analyzing the shrinkage and mechanical performance of heat-treated samples compared to non-heat-treated samples. Two parameters, heating temperature and holding time, were manipulated during the heat treatment process. The study findings can be summarized as follows.

● The study shows that the shrinkage differences of heat-treated printed parts are relatively small for all axes. Specifically, the differences between the measured and design dimensions of the printed products were relatively large, at 10.19 % and 13.78 % for tensile and flexural along the Z-axis (thickness), respectively.
● The heat treatment process can improve the mechanical strength of black carbon fiber HTPLA.
● There is no statistical evidence indicating that the two parameters, heating temperature and holding time, affect the mechanical properties of the printed parts. However, further analysis, including the interaction plot and structural analysis, suggests that these two factors might have an effect and could be considered for optimizing the heat treatment process.

## 5 Future work

Overall, this study holds the potential to advance the development and global adoption of 3D printers by offering solutions to address their limitations. Further research applications can be explored, including more comprehensive surface tests tailored specifically for black carbon fiber HTPLA, which could examine texture, porosity, and adhesion. Additionally, investigating the impact of 3D-printing angles on mechanical properties and exploring the directional influence of layers on mechanical properties could provide valuable insights. A deeper dive into correlations between layer orientation and tensile, flexural, and compressive strengths could enhance our understanding of material behavior under different stress orientations. Furthermore, future studies could examine the influence of various printing parameters, particularly the pattern used in 3D printing, and explore specific patterns that affect mechanical properties.

## Data availability statement

The authors declare that all data are included in the article, and no additional data is available.

## CRediT authorship contribution statement

**Ahmad Shah Hizam Md Yasir:** Writing – original draft, Resources, Conceptualization. **Nor Aiman Sukindar:** Writing – review & editing, Writing – original draft, Validation, Supervision, Software, Project administration, Methodology, Investigation, Funding acquisition, Formal analysis, Conceptualization. **Ahmad Afif Abdul Rahman Putra:** Writing – original draft, Methodology. **Yang Chuan Choong:** Supervision, Project administration, Funding acquisition. **Shafie Kamaruddin:** Software. **Azlan Aziz:** Project administration. **Yulfian Aminanda:** Project administration. **Mohd Hafis Sulaiman:** Supervision.

## Declaration of competing interest

The authors declare the following financial interests/personal relationships which may be considered as potential competing interests:NOR AIMAN SUKINDAR reports administrative support, article publishing charges, and writing assistance were provided by Rabdan Academy. Ahmad Shah Hizam Md Yasir reports a relationship with Rabdan Academy that includes: employment. If there are other authors, they declare that they have no known competing financial interests or personal relationships that could have appeared to influence the work reported in this paper.
